# Bilateral Patellar Tendon Rupture Following Total Knee Arthroplasty in a Patient With Alkaptonuria: A Case Report

**DOI:** 10.7759/cureus.38597

**Published:** 2023-05-05

**Authors:** Johnlevi S Lazaro, Rex Lutz, Gregory K Deirmengian

**Affiliations:** 1 Orthopedic Surgery, Jefferson Health New Jersey, Stratford, USA; 2 Orthopaedic Surgery, Rothman Orthopaedic Institute, Philadelphia, USA

**Keywords:** total knee revision arthroplasty, extensor mechanism repair, alkaptonuria, total knee arthroplasty (tka), patellar tendon rupture

## Abstract

Alkaptonuria (AKU) is a rare hereditary disorder of tyrosine degradation. The disorder is characterized by the accumulation of a pigment called homogentisic acid. Its accumulation can lead to the breakdown of connective tissue, including tendons. This report presents a 46-year-old male with a history of bilateral total knee arthroplasty (TKA) who sustained bilateral patellar tendon rupture after an acute injury. A single-stage bilateral knee revision with direct repair of the extensor mechanism augmented with Achilles allograft was performed. The procedure was successful, and the patient had an excellent post-operative outcome at one year post-operatively. This case attempts to highlight the possible complications from AKU in order to better counsel patients with this condition who are undergoing TKA.

## Introduction

Alkaptonuria (AKU) is a rare inborn error of tyrosine degradation caused by an autosomal recessive mutation in the HDG gene which encodes the homogentisate 1,2-dioxygenase enzyme. Ochronosis, a connective tissue disorder associated with AKU, is characterized by the accumulation of a pigment called homogentisic acid in cartilage, skin, and tendons [1]. 

Ochronosis can present itself in a number of ways. From a musculoskeletal standpoint, ochronosis can progress into a debilitating osteoarthritis in weight-bearing joints including the spine, knees, hips, and shoulders which is often treated with arthroplasty [2-10]. In other cases, ochronosis can lead to the breakdown of connective tissue, including tendons [11-15]. One example includes bilateral patellar tendon rupture which is presented in this case. 

Bilateral patellar tendon rupture is a rare and serious injury that occurs when the patellar tendons, which connect the patella to the tibial tubercle, become disrupted. This can cause severe pain, swelling, and difficulty walking. In the setting of AKU, the rupture is likely to be caused by the degenerative effects of this rare genetic disorder on the tendons [11, 13-15]. Treatment of patellar tendon rupture typically involves surgical repair of the torn tendons, followed by physical therapy to regain strength and mobility [16,17]. Reports of tendon injury in the setting of AKU have been described in the literature, but bilateral patellar tendon rupture status post bilateral total knee arthroplasties (TKAs) in the setting of AKU has not been described. 

## Case presentation

**Figure 1:**
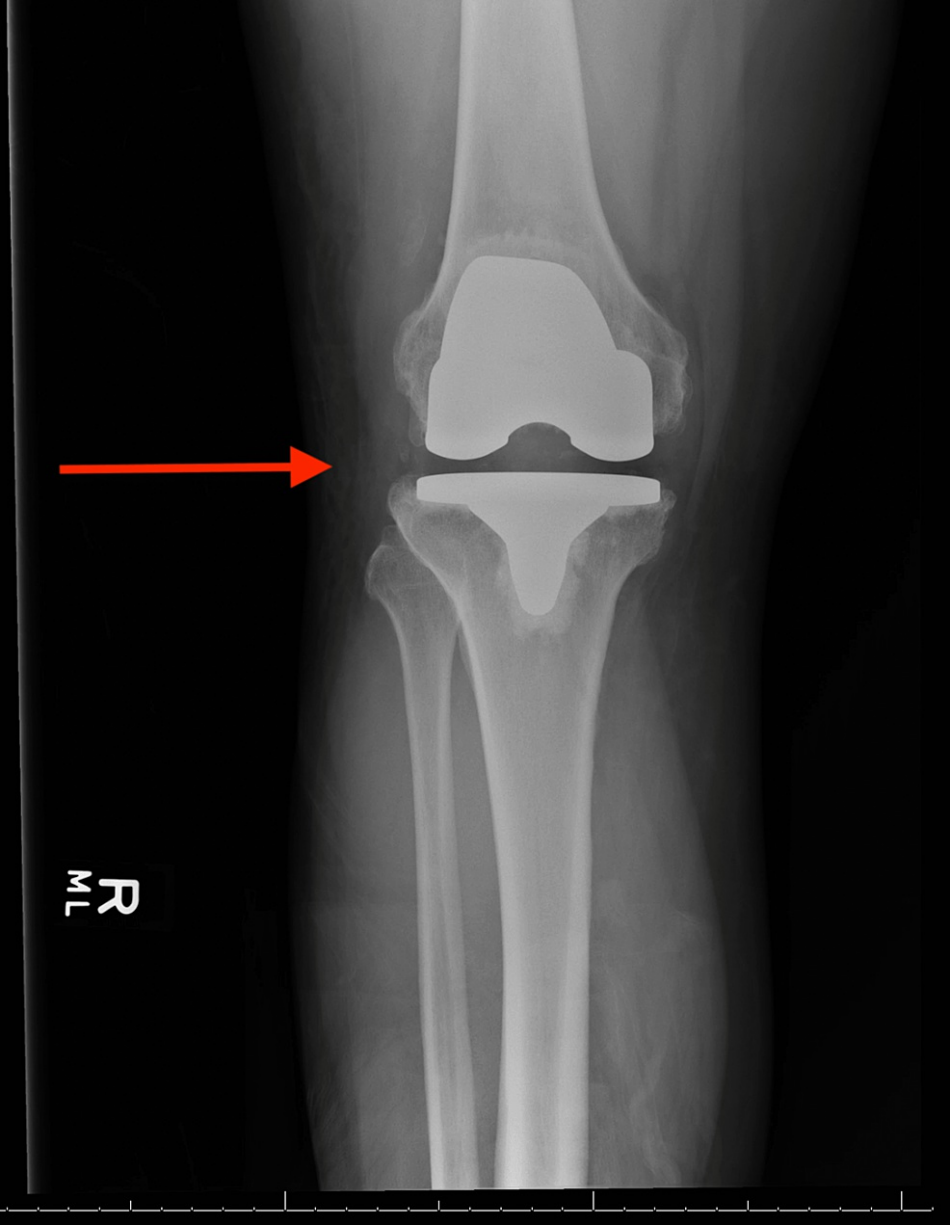
AP radiograph of the right knee Red arrow pointing to stable femoral and tibial components, no radiographic evidence of periprosthetic fracture AP: Anteroposterior

**Figure 2:**
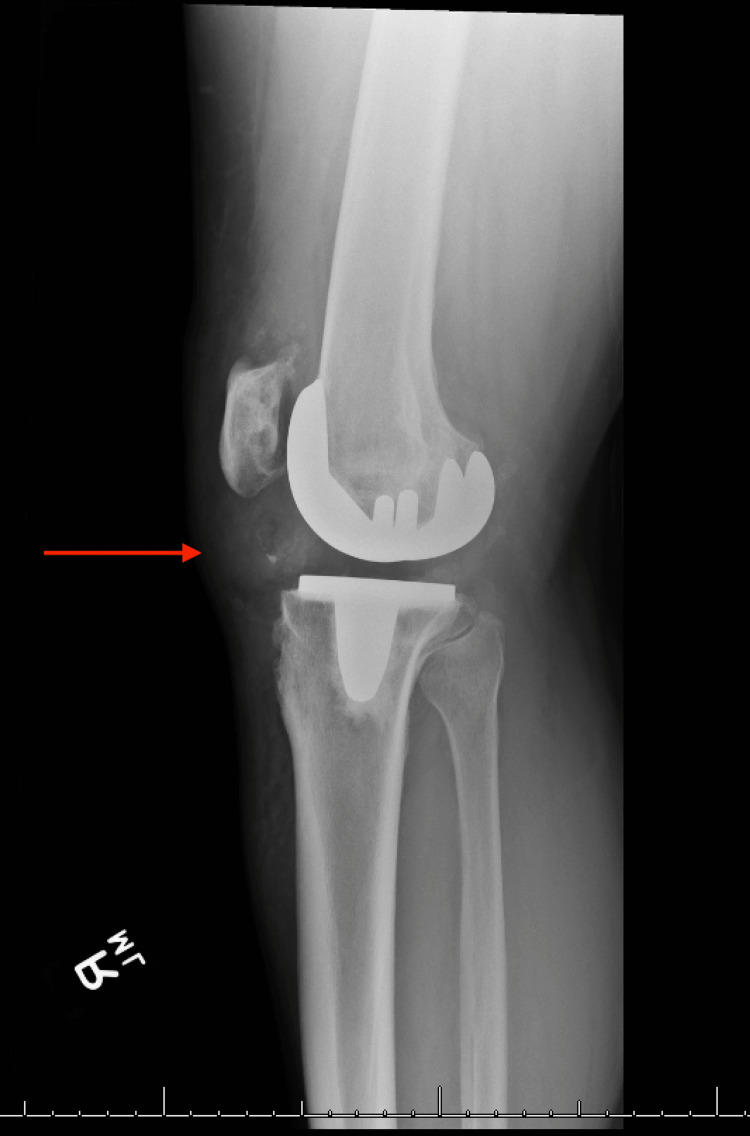
Lateral radiograph of the right knee Red arrow pointing to stable femoral and tibial components, showing a patella alta and an apparent avulsion about the tibial tubercle

**Figure 3:**
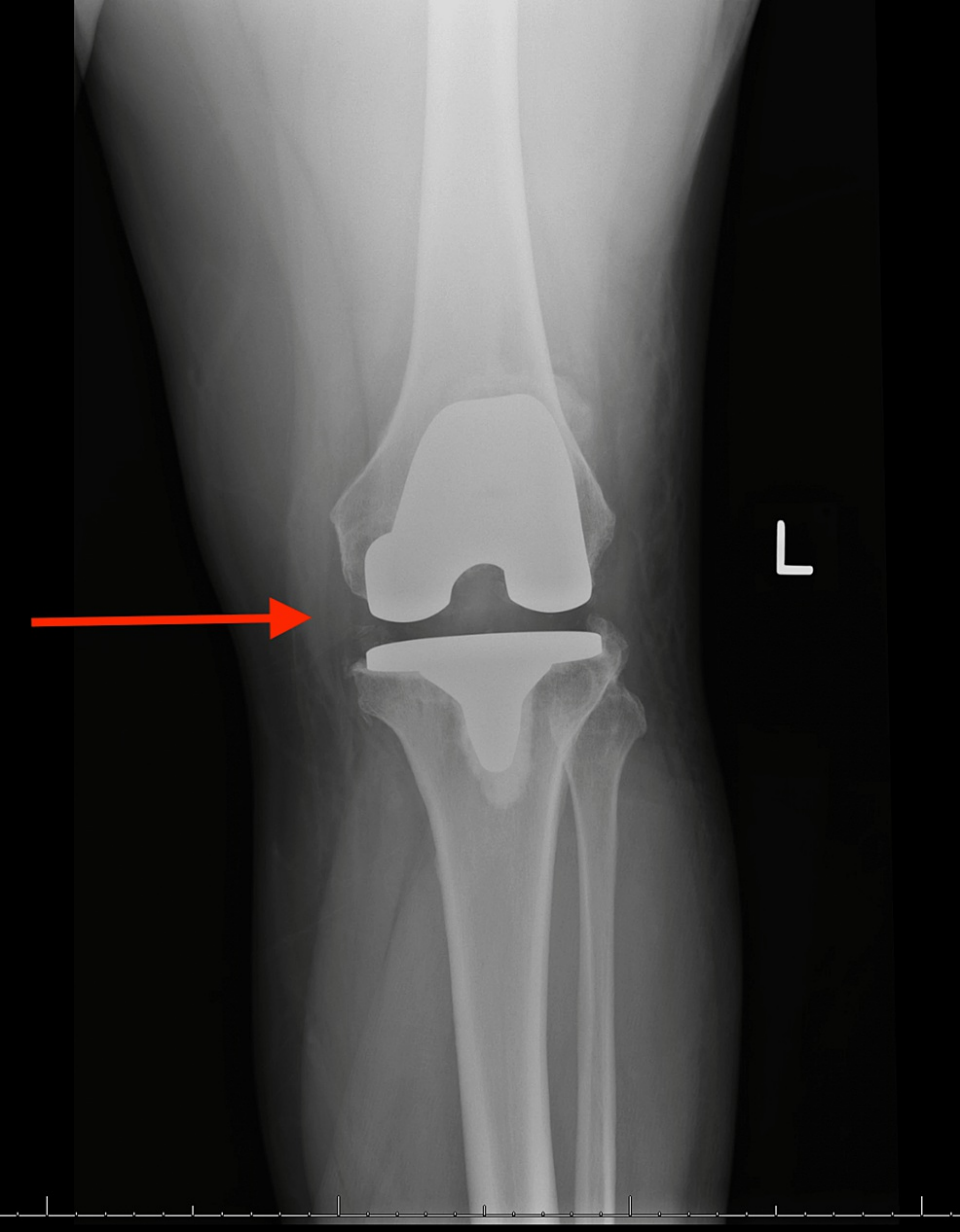
AP radiograph of the left knee Red arrow pointing to stable femoral and tibial components, no radiographic evidence of periprosthetic fracture AP: Anteroposterior

**Figure 4:**
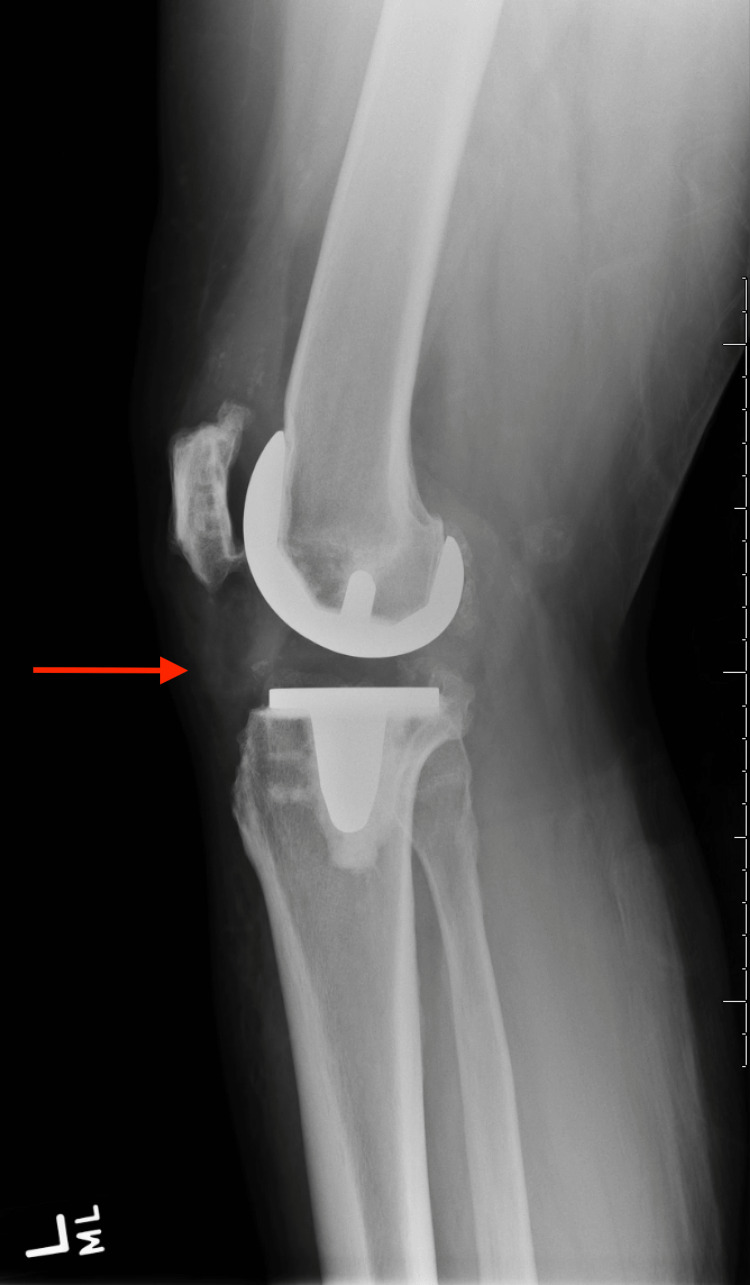
Lateral radiograph of the left knee Red arrow pointing to stable femoral and tibial components, with a patella alta

A 46-year-old male with a medical history of AKU and a surgical history of bilateral TKAs seven years prior to presentation, bilateral Achilles tendon rupture repair, and a quadriceps tendon repair was chasing a bat in his home when he felt a pop in both knees and fell to the ground. The patient was unable to get up following the injury. Radiographs were performed showing findings suggestive of patellar tendon injury (Figures 1-4).

**Figure 5:**
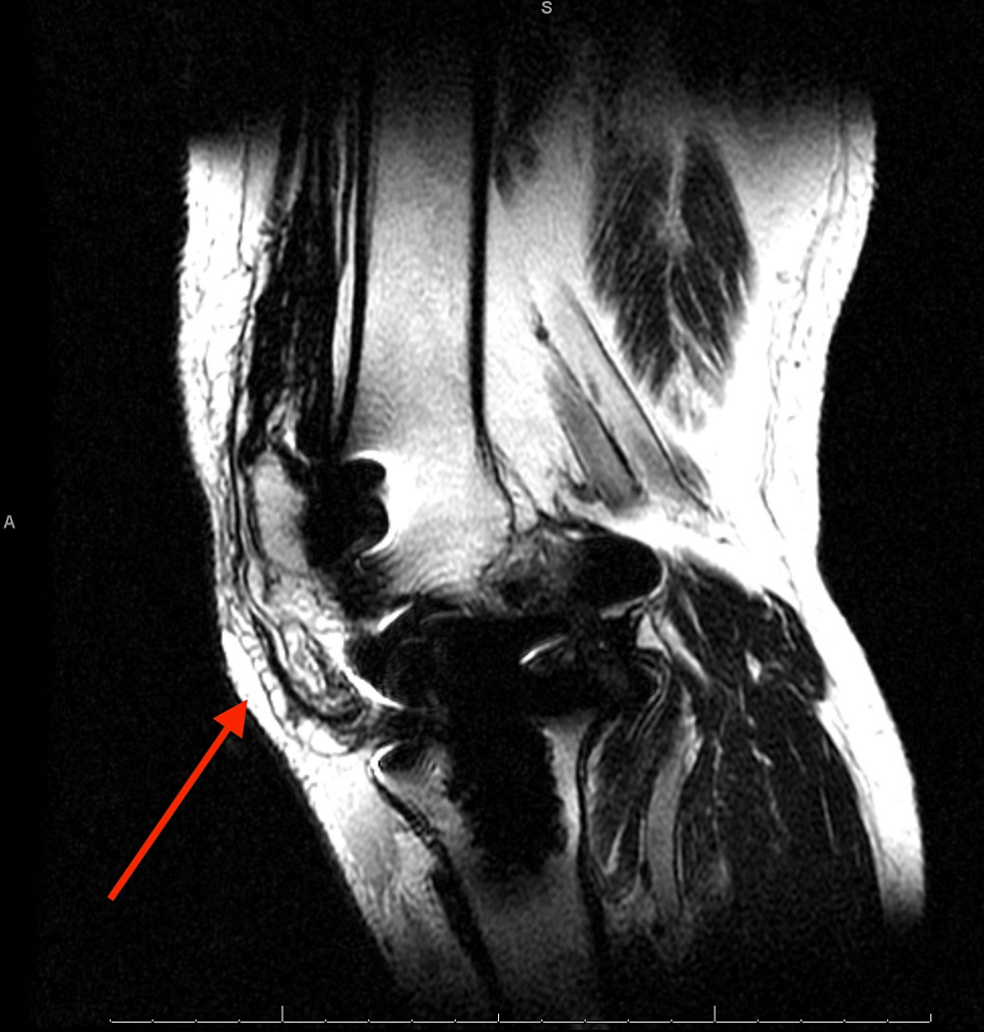
Sagittal magnetic resonance imaging of the right knee Red arrow pointing to patellar tendon rupture

**Figure 6:**
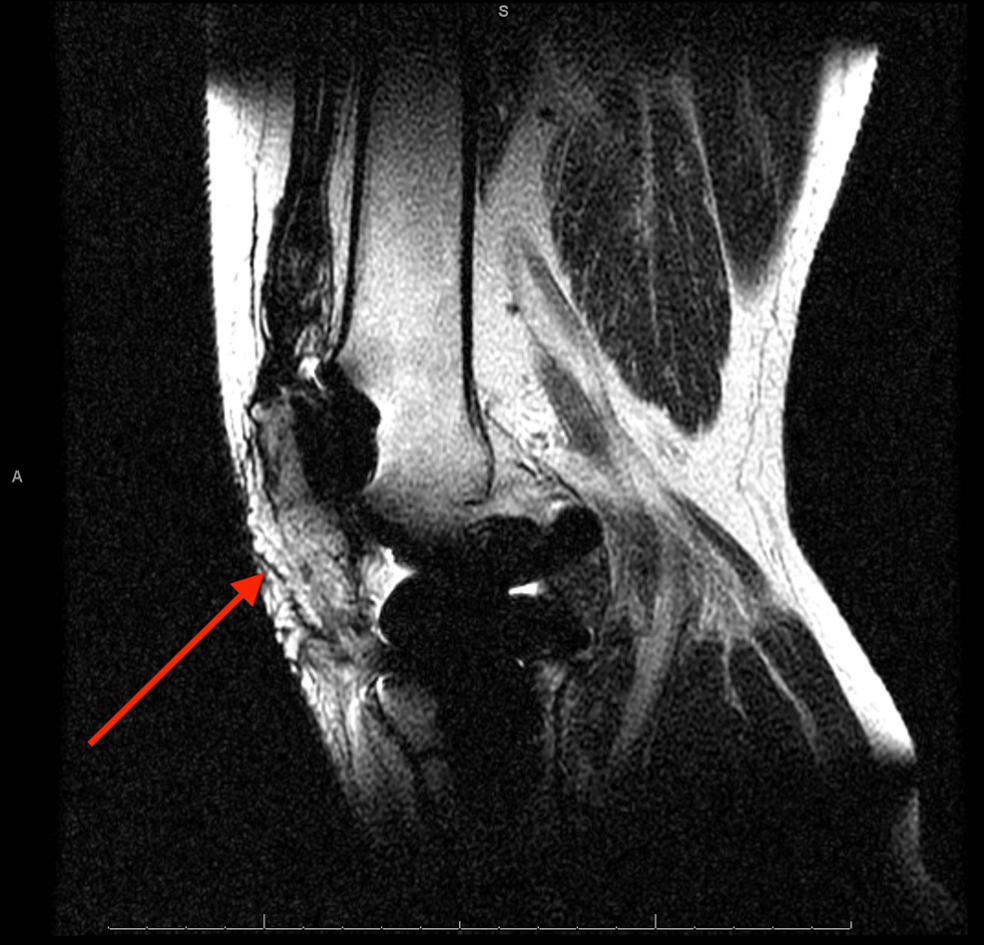
Sagittal magnetic resonance imaging of the left knee Red arrow pointing to patellar tendon rupture

MRI imaging was performed which showed bilateral patellar tendon rupture (Figures 5, 6).

He was transferred to our institution for further management where examination revealed a bilateral 60-degree lag with attempts at active extension. 

After review of appropriate studies, it was recommended that the patient undergo surgical repair. The patient was counseled on his treatment options which included both surgical and non-surgical management. The patient understood the risks, benefits, and alternatives and ultimately elected to undergo single-stage bilateral knee revision with direct repair of the extensor mechanism augmented with Achilles tendon allograft and gave informed consent. 

Surgical procedure summary 

The right knee was addressed first. After administration of antibiotic prophylaxis, the old incision was used. The patellar tendon rupture of the tibial tubercle was immediately identified. The medial and lateral retinaculum were also torn but only to the level of the ligaments without surpassing them. A medial parapatellar arthrotomy was then undertaken in order to examine the joints. The knee was exposed and irrigated. The components were found to be well fixed. The old polyethylene was removed. The ligaments were intact. Given intact ligaments and well-fixed components, the decision was made to put in a new posterior stabilized polyethylene. The knee was examined and found to be stable. 

**Figure 7:**
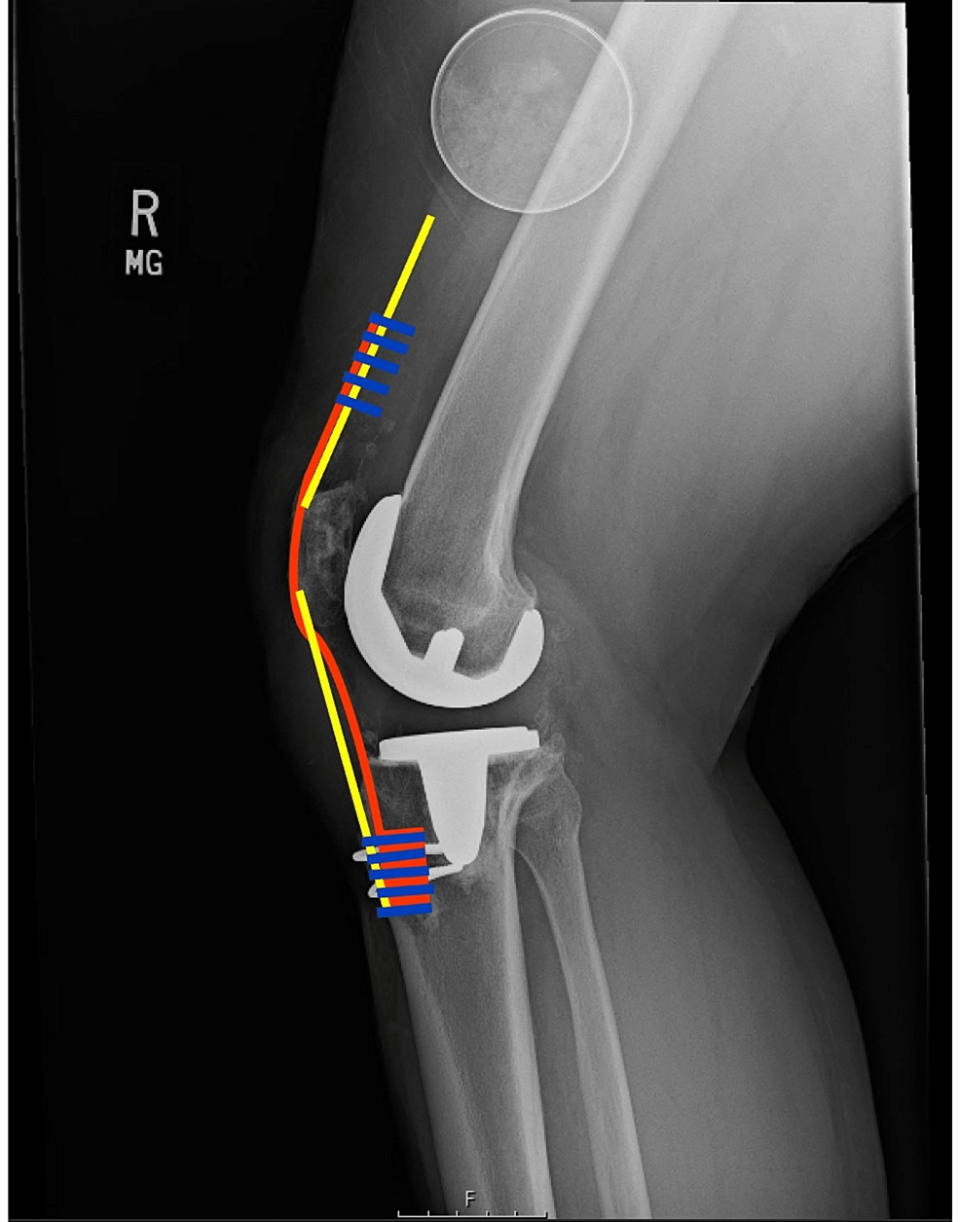
A representation of the repair projected from a lateral view Yellow represents the quadriceps and patellar tendon. Blue represents suture. Orange represents allograft

At this point in time, attention was turned to the extensor mechanism rupture. A 1 x 1 x 1 cm trough was made at the level of the native tibial tubercle. The Achilles allograft was prepared with a burr to match the trough that was created. A dovetail was made with the proximal end of the graft in order to fit in like a key and lock. The bone plug was press-fit into the trough that was created. Through drill holes, two cerclage wires were secured. The tendon was manually stressed intraoperatively and was well fixed (Figure 7). 

At this point in time, attention was turned to the patient's native patellar tendon which was ruptured. A small hole was placed at the level of the bottom of the patella through its tendon. At this point in time, two Krackow sutures were used creating four limbs of the sutures below the tendon which was secured. These four limbs were passed through drill holes and into the patient’s remaining patellar tendon at the level of the tubercle [18]. These were tied giving a repair of the patient's native tendon. Prior to tightening the sutures down, two Krackow stitches were placed within the tendon of the allograft using heavy non-absorbable sutures. These were passed from an under to over position through the hole that was made at the level of the patient’s patella. The tendon was then laid over the patient's patella and quadriceps tendon. The sutures of the native tendon were tied down in full extension of the knee. The Achilles allograft tendon was then secured to the patient’s peripatellar tissue and quadriceps tendon with sutures. Sutures were used to secure this. 

Absorbable sutures were used to repair the retinaculum, repair the medial release, and repair the remaining surrounding soft tissues, and a good closure was obtained. Irrigation was performed again, and a superficial blake drain was left in place. The subcutaneous fat and dermis were closed with absorbable sutures, and the skin was closed with staples. 

Attention was then turned to the left knee. The old incision with medical arthrotomy was used to approach the ruptured patellar tendon. The old polyethylene was removed. The components were found to be well fixed, but it was clearly evident that the patient’s MCL was also involved in the injury on this side. He was grossly instability to values stressing. Given the severe instability, the decision was made to proceed with a revision of the knee to a constrained prosthesis. Hand tools were used to remove the tibial and femoral components with minimal bone loss. A burr was used to rid the remaining cement from the femur and tibia. Reaming to a size 16 on the femur side and a size 12 on the tibial side was performed. A trial was performed with a left F LCCK (Zimmer-Biomet, Warsaw IN) femur with two 5 mm posterior augments and a 16x145 stem. On the tibial side, a size 3 tibia with a 12x145 stem was used. A size 14 LCCK polyethylene was used, and trialing with this construct showed good stability and full extension. The final TKA implants were cemented in place. 

**Figure 8:**
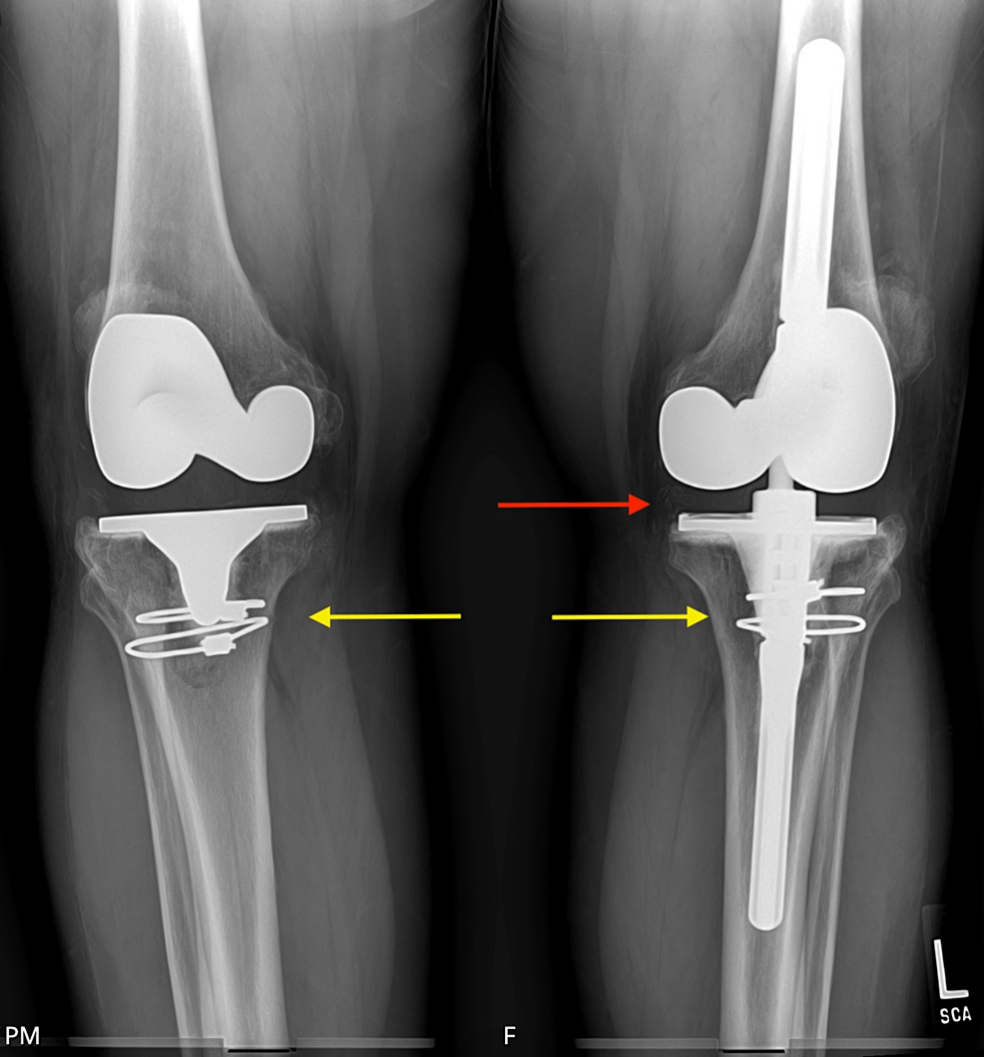
Post-operative AP radiograph of bilateral knees Red arrow pointing to the revision-constrained component. Yellow arrows pointing to cerclage wires securing bone plugs to their respective tibias AP: Anteroposterior

**Figure 9:**
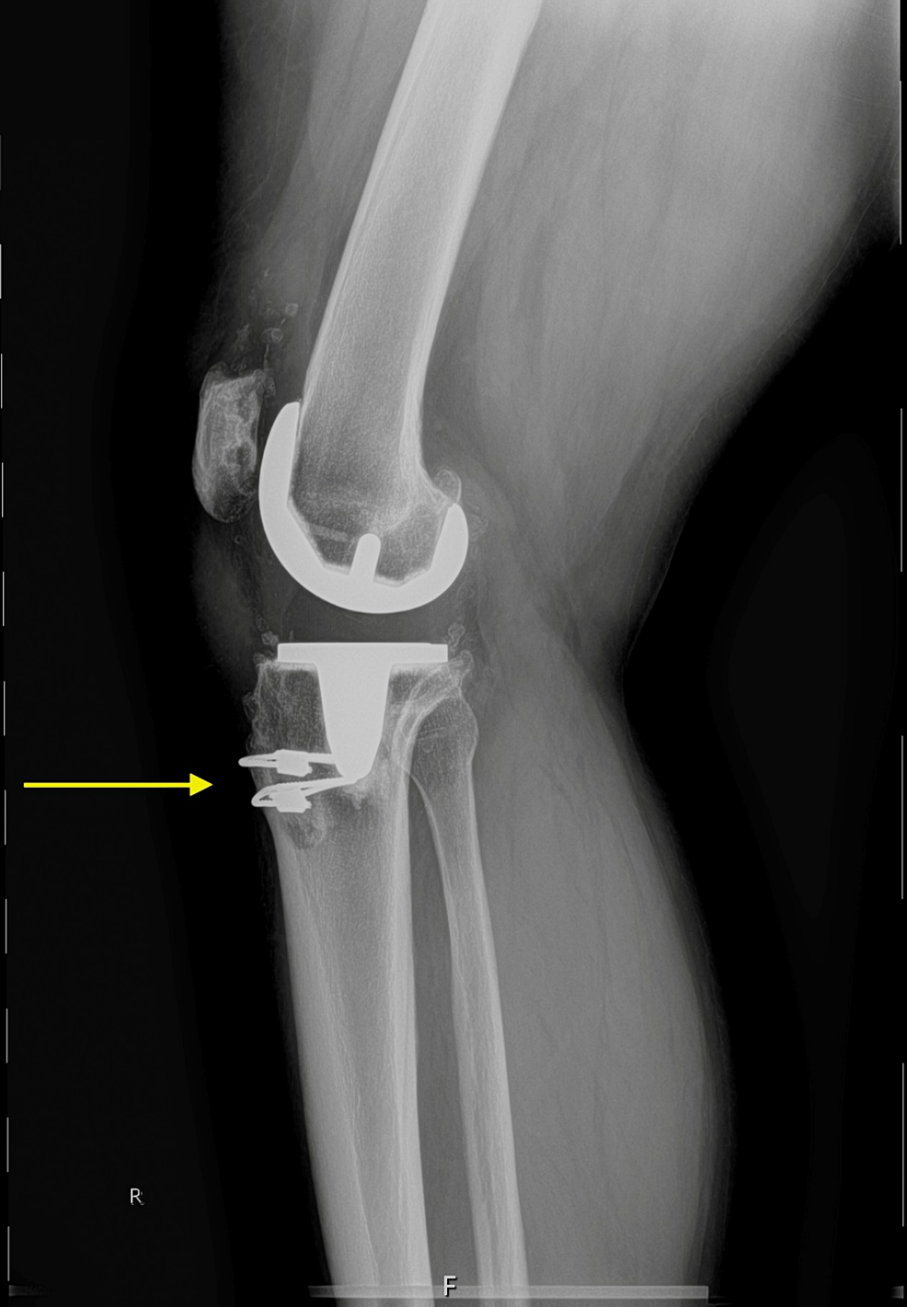
Post-operative lateral radiograph of the right knee Yellow arrow pointing to cerclage wires securing bone plugs to the tibia

**Figure 10:**
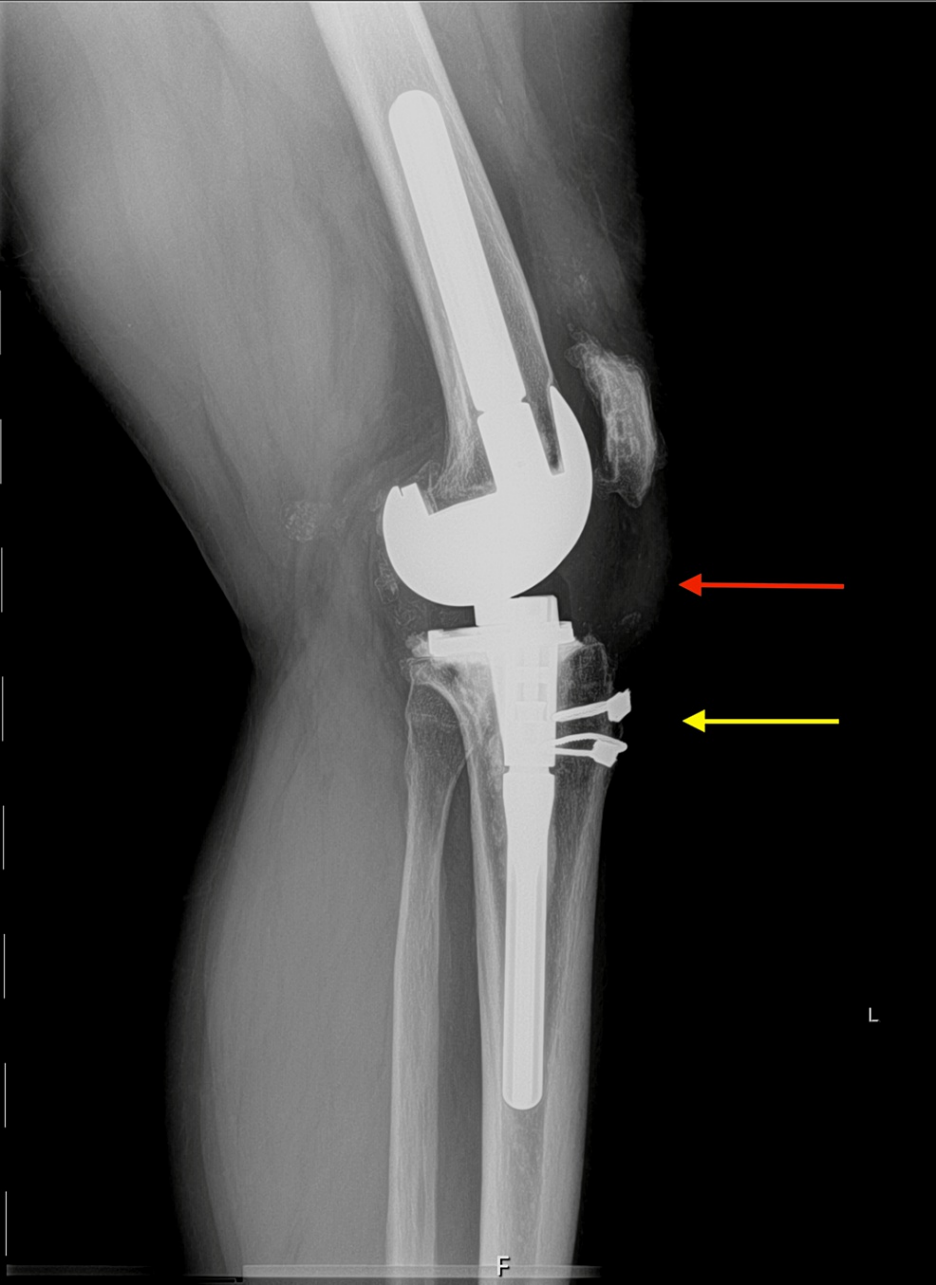
Post-operative lateral radiograph of the left knee Red arrow pointing to the revision-constrained component. Yellow arrow pointing to cerclage wires securing bone plugs to their respective tibias

The identical extensor tendon mechanism reconstruction procedure performed for the right knee was performed on the left knee in order to repair the patient's native tendon and reconstruct it with allograft. Of note, in closing the surrounding parapatellar tissues at the end of the case, it became evident due to the patient's severe nature of the left knee that a closure was tenuous at the area of the medial release due to poor tissue quality. To that end, the remnant of the allograft tendon that was cut was used as a patch over this area and secured with sutures. Hinged knee braces were then applied bilaterally (Figures 8-10). 

Post-operative course 

Post-operatively, the incision was kept clean and dry. The patient was restricted to extension for eight weeks with a hinged knee brace and non-weight bearing for eight weeks. Physical therapy was then initiated to regain functional range of motion. The patient was discharged to rehab on post-operative day 11 on Coumadin for DVT prophylaxis. By the sixth-month follow-up, the patient was walking with a cane, and his range of motion was near full extension and was able to flex the knee beyond 90 degrees. By the one-year follow-up, the patient was able to ambulate without an assistive device but would occasionally use a cane. No extensor lag was noted. 

## Discussion

Bilateral patellar tendon rupture is a rare and serious injury that can cause significant disability and impaired function. It is even more rare in the setting of AKU, a rare genetic disorder characterized by the accumulation of homogentisic acid, which can lead to the degeneration of connective tissue, including tendons. The case involves a patient with AKU status post bilateral TKA who experienced bilateral patellar tendon rupture and underwent repair. 

The incidence of patellar tendon rupture following TKA described in the literature ranges from 0.17% to 1.40% [16]. There have been case reports demonstrating spontaneous tendon rupture secondary to ochronosis [11-15]. However, to the best of our knowledge, there has been only one reported case of spontaneous patellar tendon rupture secondary to ochronosis following TKA. In a case series by Kumar et al., a patient with AKU developed a patellar tendon rupture in a native knee. Intraoperatively, the tendon was found to be avulsed from the inferior pole of the patella with an associated tear in the retinaculum [11]. The retinaculum was repaired with sutures and the patellar tendon was anchored with stainless-steel wires. Post-operatively, the patient’s knee was immobilized for three weeks before starting a gentle range of motion [11]. 

Nonsurgical management of bilateral patellar tendon rupture would require a patient to be dependent on walking aids and/or knee braces and would likely be unsatisfactory for an active patient as in our case. Surgical options include direct repair or reconstruction with allograft. Given the poor results in the literature of isolated repair of tendons, especially given this patient’s disease of his tendon and other connective tissue, the decision was made to proceed with direct repair augmented with allograft Achilles tendon reconstruction [16]. 

## Conclusions

In conclusion, bilateral patellar tendon rupture in the setting of AKU is rare but can occur and can be challenging to treat. Patients with a history of AKU who have undergone TKA may be predisposed to future revision surgeries. Our patient’s diagnosis of AKU contributed to him having very poor tendon quality which likely contributed to his bilateral tendon injuries requiring repair with allograft. While his post-operative course was successful, there are serious risks of knee stiffness, extensor lag, and infection with repair. It is important to counsel patients with a history of AKU about the possibility of tendon injuries, which may require future operations. 
